# Incorporating Quantitative Literacy into a T32 Retreat: Lessons and Considerations from Experience

**DOI:** 10.1523/ENEURO.0027-25.2025

**Published:** 2025-06-12

**Authors:** Adam A. Hall, Jenna R. McGrath

**Affiliations:** Marion Murray Spinal Cord Research Center, Department of Neurobiology and Anatomy, Drexel University College of Medicine, Philadelphia, Pennsylvania 19129

**Keywords:** data analysis, data replication, graduate students, quantitative literacy, retreats, spinal cord injury

## Abstract

Regardless of discipline, quantitative literacy is a critical component of any scientist's skill set. A recent push from the NINDS has focused on enhancing and maintaining this expertise in trainees to enhance scientific fluency and to combat the reproducibility crisis. T32-funded programs often include off-campus retreats, providing opportunities to integrate a quantitative literacy component, or thematic focus. Here we will discuss the lessons and considerations learned from organizing a retreat focused on quantitative aspects of diagnostics for spinal cord injury. Survey results regarding retreat events and workshops reveal elements that were perceived to be successful by attendees. Events developed with active learning that focused on collaborative problem-solving and cross-discipline quantitative measures were well received by trainees. On the other hand, lectures and panel discussions were found to be less effective in boosting long-lasting improvements in quantitative literacy. Taken as a whole, these experiences from incorporating quantitative literacy into a T32 retreat offer strategies to consider when designing coursework or events focusing on this topic.

## Significance Statement

Recent reports regarding the crisis in replicating scientific findings have led to a concerted effort by the NIH to enhance quantitative literacy. Development of T32 departmental retreats with an emphasis on active learning to shape statistical and experimental design standards are discussed. Attendee survey results following the retreat strongly suggest that future programming should emphasize collaborative exercises focusing on quantitative literacy in lieu of passive lectures.

## Introduction

### The importance of quantitative literacy and scientific fluency

Quantitative literacy is the ability to interpret numeric information by using the skill of mathematical thinking (Speth et al., 2010). Quantitative literacy focuses on comprehending numerical information in daily experience, with the ability to engage with analytical questions as they arise in multiple contexts. Efforts in defining quantitative literacy for training purposes have focused on fostering skills in data analysis, data management, and statistical justification of approach and interpretation. Beyond discrete examples focusing on data management, training in quantitative literacy enhances capabilities in strategically evaluating new information and using the results to support conclusions. This is central to capturing relationships between variables, a skill essential for the development of testable hypotheses. Quantitative literacy is more than just understanding statistics, but applying knowledge to evaluate and reason with data-based claims.

Scientific professions universally require skills in organizing information and critically analyzing claims tied to real-world problems. Quantitative literacy helps ensure a standardized means in portraying findings across disciplines, allowing for a shared language between all scientists that facilitate easier engagement between fields. This provides the basis for scientific fluency, which stems from comfort in experimental design and quantitative literacy. Scientific fluency empowers researchers to interpret data and experimental design that is not closely related to their field of expertise. This often leads to fruitful collaborations, providing an opportunity for outside review and innovation (Prescott and Huang, 2023).

The ability to communicate quantitative literacy skills is as important as the skills themselves. Comfort in discussing these topics leads to a mindset that recognizes the world as a collection of quantitative relationships and a willingness to engage, rather than avoid, quantitative information (Roohr et al., 2014). Quantitative literacy and scientific fluency have become increasingly relevant given the active replication crisis in biomedical research. NINDS-T32 sponsored departmental retreats designed to focus on quantitative literacy present opportunities to reinforce quantitative and experimental skills.

### The ongoing replication crisis, a problem of training

Training both students and faculty in quantitative literacy provides them with crucial skills for conducting quality science. This has become increasingly relevant as the replication crisis within STEM fields has been brought to light. Recent worrying figures in reference to scientific rigor and replication clarify the need for intervention across scientific disciplines. A survey conducted by *Nature* found that >70% of 1,576 researchers have tried and failed to reproduce another scientist's experiments. Perhaps more disturbing, more than half of the respondents failed to replicate their own findings (Baker, 2016a). Emblematic of these ongoing issues, many studies have shown that >50% of biomedical science papers fail to replicate (Begley and Ellis, 2012; Baker, 2016a; Korbmacher et al., 2023), with the expected cost to taxpayers being as high as 28-billion dollars per year (Freedman et al., 2015). Without an emphasis on replication and experimental rigor, trainees are left in a position to prioritize other experimental concerns, resulting in recurrent lapses in replicable papers.

### Departmental retreats offer more than a respite

Active learning can turn the often-perceived chore of quantitative literacy education into an opportunity to enrich other areas of study. Active learning emphasizes ongoing participation in the learning process. These activities often require self-reflection and critical thinking, enhancing the attention and buy-in from trainees (Prince, 2004). Active learning has been shown to lead to better recall and mastery of quantitative literacy concepts when compared with passive practices (Speth et al., 2010 ). One tool to drive active learning and provide a stand-out experience for trainees is a department-wide retreat focusing on one scientific topic with programming infused with lessons on quantitative literacy and scientific fluency. These retreats break up monotony and provide memorable opportunities to integrate quantitative literacy skills.

To provide insight into how to develop engaging and effective T32 retreats, below we spotlight programming and lessons from the 2023 Drexel University, Marion Murray Spinal Cord Research Center, departmental retreat, Diagnostic Tools for Spinal Cord Injury Treatment.

#### Preprogram events

The first event of the retreat emphasized an active learning objective, where trainees were asked to generate prompts associated with ongoing quantitative literacy issues in their research. These often focused on discussing the statistical method best fit to the current experiment but also included possible controls or suggestions for future experiments. This event was particularly helpful as it exposed trainees to techniques and common questions found in labs they otherwise would rarely interact with. This session left trainees with new ways to consider approaching their own data analysis and to appreciate how other techniques could enhance their research.

Following the data troubleshooting workshop, trainees and retreat speakers were invited to a poster session where trainees collaboratively displayed their work. This session provided an opportunity for trainees to engage directly with invited speakers to discuss nuances in the development of their research. Presenting these posters as a group allowed trainees to address lab-wide issues with the visiting expert, who could provide novel troubleshooting efforts from an outside perspective.

#### Program events

The day of the retreat consisted of two keynote speeches, mixed with three breakout room sessions, and finished with a panel discussion led by the keynote speakers and breakout room leaders. In line with the theme of the retreat, we invited speakers that could provide insight on different aspects of utilizing diagnostic tools for spinal cord injury. Our first speaker, Dr. Adam Ferguson, is an expert in behavioral neuroscience who studies mechanisms of recovery after neurological trauma, such as spinal cord injury. In addition, he serves as the founding principal investigator and codirector of the International Open Data Commons for Spinal Cord Injury. This Open Data Commons was founded to serve as a repository where members can store and access data. Throughout his talk, he provided examples of how data sharing through a large database can be used to empower future treatments for spinal cord injury. Our second speaker, Dr. Keith Tansey, has an M.D., Ph.D. in neuroscience and is board-certified in neurology with a subspecialty in spinal cord injury. He is also a past president of the American Spinal Injury Association which has previously developed the International Standards for Neurological Classification of Spinal Cord Injury (ISNCSCI) exam. This exam has been widely used in the clinic to grade the severity of a spinal cord injury. Both talks were presented in a traditional format that allowed 45 min for the talk and 15 min for questions. This format relies on passive learning and assumes that the participants will absorb the information without direct involvement.

To introduce interactive components to the retreat, we arranged three breakout rooms that attendees cycled through. Each breakout session focused on a specific topic and was facilitated by a breakout room leader with expertise in the respective topic. The three breakout rooms were “Pain Assessment in Spinal Cord Injury” led by a Drexel principal investigator who specializes in spinal cord injury related pain research, “Spinal Cord Injury Rehabilitation from a Clinician's Perspective” led by a clinical researcher at Thomas Jefferson University with a doctorate in physical therapy and “Validating Diagnostic Tools” led by our T32 quantitative literacy expert and a T32-funded trainee. The breakout leaders were given full control of how they wanted to organize their room which resulted in a variety of presentation styles and levels of discussion. One of the breakout leaders opted for a more formal presentation style, with opportunities for attendees to ask questions throughout the presentation. The other two breakout rooms divided participants into small groups and provided them with a discussion topic. The participants in the breakout rooms were composed of trainees, principal investigators, postdoctoral researchers, and technicians, allowing for multiple perspectives on the various topics. After allowing for adequate conversation in small subgroups, discussion continued with the entire breakout room. Each room touched on the use of different tests used to evaluate function after a spinal cord injury. Each of these tests require standardization that can be accomplished with different methods of quantification. This was specifically focused on in the “Validating Diagnostic Tools” breakout room where discussions revolved around various predictive measures that could be used for evaluating functional outcomes after a spinal cord injury. This room highlighted the importance of quantitative literacy when evaluating and establishing a universal predictive measure to be used in the clinic.

Lastly, we ended the retreat with a panel discussion designed for the attendees to reflect on what they had learned from the day and ask any remaining questions to the panel with various areas of expertise. From this, questions could be answered through the lens of a researcher, a clinician, and a statistician. The panel also relied on active participation from the audience but was not held in the same type of intimate setting as the breakout rooms. Due to this, we found that it was more difficult to promote the same type of organic and vibrant discussions.

#### Postretreat analysis

**Figure 1.**
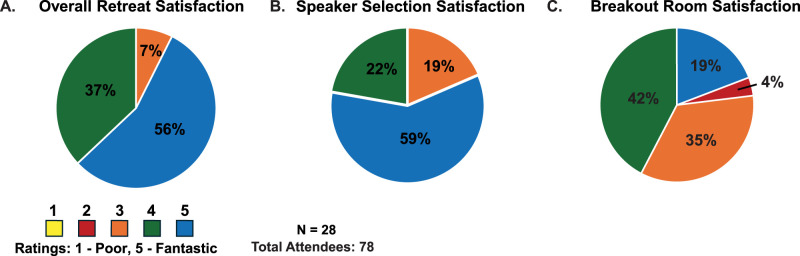
Postretreat attendee survey results. Attendees were asked to complete a survey where they rated (***A***) overall retreat satisfaction, (***B)*** speaker selection satisfaction, and (***C***) breakout room satisfaction on a scale from 1 (yellow) to 5 (blue), with 1 being poor and 5 being fantastic. Out of the 78 attendees, we have survey results from 28 of them.

Following the event, all attendees were asked to complete a short questionnaire to assess the quality of the retreat. The questionnaire consisted of rating different aspects of the retreat from 1–5 (1 = Poor and 5 = Fantastic), as well as open response questions to obtain more detailed feedback. We asked questions regarding both the logistical aspects and the content of the retreat. The results from this survey demonstrated that the vast majority of attendees had an overall positive experience (Fig. 1*A*, 7% scored a 3, 56% scored a 4, 37% scored a 5) and felt the chosen speakers matched the needs and expectations of the retreat. While speaker feedback was positive overall (Fig. 1*B*, 19% scored a 3, 22% scored a 4, 59% scored a 5), when asked for input on future speakers, some shortcomings became evident. Attendees pointed out that future years would benefit from a more diverse group of speakers. There were also recommendations toward selecting speakers with perspectives outside of our research center, including those involved in clinical trials and human research. The most constructive criticism we received referenced the breakout sessions (Fig. 1*C*, 4% scored a 2, 35% scored a 3, 42% scored a 4, 19% scored a 5). Some felt that the organization of the breakout rooms could be improved by providing the leaders with a list of discussion points related to the topic. This can be achieved through objective focused discussions prior to the retreat with leaders or provision of topic-centered handouts that help facilitate efficient breakout rooms. Lastly, we inquired about what the attendees disliked about the retreat and/or points for improvement. One of the most frequent comments focused on the lack of participation from those living with a spinal cord injury. Many people felt that individuals living with a spinal cord injury should play a more active role in the retreat, providing insights into lived experience and research trajectories. The survey demonstrated that the retreat was widely viewed as being successful, with few, but critical, points for improvement.

#### Best practices and guidance for retreat planning

Our experience in planning a retreat gives us valuable insights into effective practices and potential challenges and pitfalls. The goal of the retreat was to set aside designated time for trainees and faculty to have conversations about the diagnostic tools currently being used in spinal cord injury treatment and to incorporate quantitative literacy throughout these discussions. Through our efforts, we learned the following lessons (summarized in Table 1):

**Table 1.** Retreat summary, best practices, and pitfalls

Category	Description
Objective	(1) Ensure engagement
	(a) Active Learning
	(2) Specifically crafted activities
	(a) Emphasis on quantitative literacy
Action	(1) Buy-in through personalized project
	(a) Experimental design and statistics workshop
	(b) Poster presentation with quantitative emphasis
	(2) Retreat activities
	(a) Alternate active learning with passive lectures
	(3) Reflection and follow-up
	(a) Postretreat trainee meeting
	(b) Satisfaction survey
Best practices	(1) Breakout rooms
	(a) Enables direct engagement between speakers and trainees
	(b) Provided safer space for involvement of everyone
	(2) Breakup of passive and active learning
	(a) Schedule lectures before and after breakout rooms with a small group format
Potential pitfalls	(1) Overwhelming and/or impromptu activities
	(a) Preretreat data workshop requires a lot of organization and familiarity between trainees and the quantitative literacy expert
	(2) Breakout rooms functioning as lectures
	(a) Breakout rooms not designed around active learning principles were rated much lower with less buy-in
	(3) End of retreat panel discussion
	(a) Minimized audience participation
	(b) Incentivized only the most senior/extroverted to speak
	(4) Lack of diversity
	(a) Retreats missing gender, racial, and patient diversity lack critical perspectives

A summary of the objectives we had for the retreat, the actions we took to achieve those objectives, and what we found to be the best practices and potential pitfalls.

### Design workshop content to benefit the entire audience

We found that the questions trainees submitted ahead of the workshop were often highly detailed, limiting the scope of the application. This level of detail made it difficult for the statistician to constructively answer while benefiting the entire audience. While we found that asking trainees to submit questions prior to the workshop was effective in getting them to think about how to apply statistics to their research and identify challenges, the session was not as successful in addressing questions tailored to specific research projects. This hindered the ability of the workshop to convey nuanced concepts of quantitative literacy relevant to the entire audience. Instead, sufficient time should be dedicated prior to the event to organize the material, ensuring that themes from the questions are effectively addressed. This includes asking students to submit questions over a week in advance of the workshop and working to extract the broader topics from the specific questions. These broader topics can then be provided to the workshop leader so they can prepare how to best address the material.

### Small group sessions are most effective at promoting broad participation

Based on our experience facilitating small group sessions and observing the panel discussions along with the question and answer sessions after the keynote speeches, it was clear that small group sessions generated significantly more discussion. In particular, our breakout rooms consisted of 20 or fewer participants. In two of the breakout rooms, attendees were split into 3–4 subgroups and given an activity to discuss. For example, one breakout room gave each subgroup a handout detailing a various aspects of diagnostic tools used in spinal cord injury clinical practice, biochemical, imaging, and physical testing. Subgroups were asked to discuss the relative strengths and weaknesses of each procedure and how quantitative literacy practices could impact the efficacy of each set of techniques. This objective-centered approach noticeably enhanced audience participation. When compared with the breakout room that was not split into subgroups and instead given a lecture on clinical rehabilitation strategies, audience participation and satisfaction was lower. Participation began to decrease as the size of the room surpassed 15 people. This was evident during large group discussions with >70 attendees. The people engaging with larger group discussions, such as the panel discussion, were overwhelmingly professors or the trainees organizing the retreat. Trainees later reported that small group discussions minimized the anxiety associated with speaking out. These groups were often structured in a way that encouraged discussion, making involvement from all attendees an expected part of the experience. From our experience it is imperative to remove barriers and found that creating an environment that reduces anxiety around speaking out is key to fostering greater attendee engagement. These findings emphasize the importance of planning structured activities for small groups that minimize the chance for distraction and increase the ease of audience participation.

### Emphasize inclusion of diverse speakers and prioritize lived experience of relevant patients

One of the most common criticisms of this retreat was the lack of inclusion of diverse speakers from a gender, socioeconomic status, or racial background. The lack of diversity limited the perspectives that our presenters were able to bring to the event. Importantly, while we had an attendee living with a spinal cord injury, they were not a focal point of the programming, and their perspectives were not shared with the entire audience, limiting its impact. This lapse disconnected the stakes of the research from the discussion. A major factor contributing to the lack of available treatments is the chronic presence of irreproducible science publications. This was exemplified by Amgen failing to reproduce 47 of 53 papers they thought could lead to treatments (Baker, 2016b). With the various pressures to publish well, it is easy to disconnect from the stakes associated with the work preclinical researchers do. Exposure to individuals early and often help reify the stakes of rigorous science. This emphasizes the importance of including individuals with the disorder in any retreat focused on its research. Their involvement goes beyond providing personal testimonials but also ensures that the audience gains a deeper understanding by integrating their hopes and real-world needs into their research objectives.

## Discussion

### Incorporating quantitative literacy into a departmental retreat

We achieved our main goal of incorporating quantitative literacy into all aspects of the retreat. In addition to the formal program events, we implemented new preprogram events focusing on quantitative literacy. Our data troubleshooting workshop primed the trainees for discussions that would be held the following day and gave them the opportunity to ask statistical questions pertaining to their specific research projects. Our survey demonstrated that trainees found this “workshop” style of event to be particularly useful, claiming that it accompanied an “excellent statistics talk.” Following the workshop, a trainee poster session was held to provide the chance to discuss their ongoing research projects with the guest speakers, as well as other members in the department. While quantitative literacy can be brought up in these conversations, future retreats will implement a system for trainees to highlight aspects of their poster where they demonstrated apt quantitative literacy. These features will align with the practices associated with replicative success in the literature and are points of emphasis with the NIH. Examples of statistical preregistration, equal sex representation in animal subjects, randomization, and blinded experimental design, among other factors, will be noted. The use of these rigor icons has begun to be piloted at scientific conferences and is encouraged by the NIH (Silberberg et al., 2017).

### Active versus passive learning

We arranged the retreat to include both passive and active learning. This allowed our attendees to receive valuable information from our keynote speakers’ presentations and delve into deeper discussions in the more relaxed environment of the breakout rooms. Active engagement with quantitative literacy techniques has been shown to solidify those practices better than passive lessons about them (Markant et al., 2016). Integrating aspects of repetition, critical reflection, collaboration, and directed problem-solving have been shown to be more effective at long-term retention than passive lectures (Prince, 2004; Markant et al., 2016; Kooloos et al., 2020). A retreat emphasizing actively learning quantitative literacy practices may instill these skills faster than modules that trainees passively watch, or skip entirely, to fulfill funding requirements. Aligning with previous literature, our survey revealed that attendees found instances of active learning to be more productive and valuable. They felt that this was more easily fostered in breakout rooms without formal presentations. Attendees rated those sessions as “interactive” and “unique” and enjoyed the discussions that were possible with smaller groups. On the other hand, components utilizing passive learning require future improvement. Attendees reported that the panel discussion was not very effective and felt more like a “time-filler.” While we encouraged questions and started the panel with some questions of our own, there seemed to be a lack of enthusiastic involvement from the attendees. Similarly, some felt that not much was accomplished in the breakout room with a formal presentation and emphasized that future topics should promote active discussion for both trainees and PIs. Both points suggest that active participation can be fostered in smaller and more casual environments. This will be heavily considered and implemented whenever possible in future retreats.

### Quantitative literacy to stem the replication crisis

When trainees are not well versed in quantitative literacy or scientific fluency, researchers can become blind to lapses in experimental and statistical design. This is thought to be one of the primary causes of the replication crisis in the STEM fields (Baker, 2016a). Academics, funding agencies, and the NIH (Collins and Tabak, 2014) have taken these issues seriously and begun to address them. Numerous initiatives have been created by the NIH and top-tier journals to find and address blind spots in experimental rigor and have begun to yield positive results (Korbmacher et al., 2023). This has included a shift to in-person events that highlight quantitative literacy and scientific fluency for trainees, such as T32 retreats. The NIH has also instituted numerous requirements, such as a series of modules designed to amplify data reproducibility, which trainees and professors would need to complete as a requisite for grant funding (Clearinghouse for Training Modules to Enhance Data Reproducibility, 2023). This program focused on group blinding, randomization, sex as a biological variable in both animal and in vitro research, along with validation of key resources. Recent studies have shown that when instituting rigor-enhancing practices, it is possible to broadly replicate findings (Evans et al., 2023). Ironically, one highly publicized publication that emphasized that it is possible to replicate social science findings was retracted due to lack of experimental transparency, preregistration, and incomplete data reporting (Protzko et al., 2024a,b). This retraction makes it clear the need for quantitative literacy training, rigorous adherence to data stewardship, and scrupulous attention to detail.

Importantly, while the lack of statistical education contributes to the replication crisis, many other factors play a role. The threats of dark data, publication bias, inadequate reporting standards, and the unrelenting pressure to publish often in high-impact journals can all lead to issues with reproducibility. Taking an active approach to educating trainees on these issues can help curb many of the habits undermining the replication crisis.

Dark data is any data not used or excluded from analyses, leaving blind spots in publications that fit an expected narrative more cleanly (Kim, 2024). Recent studies have found that >50% of biomedical research and possibly 80–90% of clinical research output is thought to be dark data (Hand, 2020; Null Hypothesis Initiative, 2022), with others finding this trend intensifying over time (Ornes, 2021). The tremendous amount of dark data skews publicly available datasets, compromising the effectiveness of meta-analyses, and wastes resources on investigations that have already been found to be fruitless (Murphy and Thomas, 2024). Educating trainees on what dark data is, minimizing the stigma of publishing data that was unexpected, or contrary to the original hypothesis will be critical to combating these trends. These kinds of hands-on retreats represent a powerful opportunity to instill these lessons early.

The collection of dark data can lead to publication bias, which is defined as the failure to publish results based on their direction or strength (Nair, 2019). Researchers may feel less compelled to publish negative results since they may be deemed as a failure. The importance of publishing and reporting all data, even negative data, was a focal point of Dr. Adam Ferguson's lecture. He spoke about his experiences using machine learning to analyze previously recorded data from spinal cord injury patients. This analysis revealed mean arterial pressure as a predictor for neurological recovery after spinal cord injury (Torres-Espin et al., 2021). This finding was possible because of the detailed operating room records available. Dr. Ferguson encouraged the use of the Open Data Commons to provide a repository for preclinical data. Publishing and reporting all data, including negative data, can allow for the analysis of large data sets, which can reveal new trends and perhaps, similar to Dr. Ferguson's findings, reveal new biomarkers.

Inadequate reporting standards can also contribute to the reproducibility crisis. As reporting standards become less enforced, the natural impulse to include fewer details becomes stronger. Multiple standards have been introduced since 2010 to enhance transparency in experimental design and data reporting (Wilkinson et al., 2016; Council, 2019). Researchers have struggled to abide by these standards with one study finding important factors such as sample size justification (90%), randomization (57%), and blinding (77%) being largely omitted from published work (Reynolds and Garvan, 2021). However, there is some cause for optimism, as another article found that adherence to reporting standards associated with variables that could affect study results rose from 63 to 73% (Gopel and Burggren, 2022) over a 3 year period. This suggests that given enforced standards and education about what constitutes critical information, researchers can generate more transparent work, hopefully leading to stronger reproducibility rates.

This pressure can create a breeding pool for scientific misconduct. Scientific misconduct has been found in 1–3% of published works in the United States and Europe (Bauchner et al., 2018; Xie et al., 2021), but in situations where the pressure to publish is more intense, misconduct runs rampant. Research institutions in China were previously organized through a “papers-only” rule where employment was directly tied to publication rates, and these incentives led to scientific misconduct being reported by up to 33% of these researchers (Qiu, 2010). Even with aggressive changes to the policy (Li, 2020), widespread retractions have still been reported in 2024 (Mallapaty, 2024). This makes it clear that once researchers’ livelihoods are dependent on how quickly they can publish, the stronger the trend toward scientific misconduct becomes. It is substantially easier to maintain a transparent culture that hedges against scientific misconduct than to combat an entrenched set of incentives that undermine reproducibility. This is where early education in retreat-like settings can consistently maintain the importance of quantitative literacy and the rigor that comes with it.

These reports demonstrate the complexity of the replication crisis as a multifaceted issue. Many factors must be addressed to combat this issue, with one being adequate quantitative literacy training. It is clear that quantitative literacy training enhances rigor and can reduce issues with replication. Though progress has been made by instituting these stipulations, more effort needs to be applied to ensure the underlying lessons are integrated at all levels.

## Conclusion

Retreats present a unique opportunity to expose a diverse set of trainees and principal investigators to novel topics and questions. Therefore, it is an opportune time to incorporate quantitative literacy into themes that still capture the audience's overall interests. From our survey and other informal feedback, incorporating elements with active learning allowed us to successfully integrate quantitative literacy into this retreat. Active learning promoted attendee engagement, increasing the likelihood that the lessons left a lasting impact on the attendees. Our attendees greatly appreciated the effort that went into planning the retreat, and the majority felt as though it was a productive day of discussion and learning.

## Synthesis

Reviewing Editor: Jennifer Dulin, Texas A&M University Decisions are customarily a result of the Reviewing Editor and the peer reviewers coming together and discussing their recommendations until a consensus is reached. When revisions are invited, a fact-based synthesis statement explaining their decision and outlining what is needed to prepare a revision will be listed below. The following reviewer(s) agreed to reveal their identity: Tuan Bui.

Both authors and the editor agree that this manuscript has potential to improve training activities through emphasis on active learning rather than passive learning. Revisions include accounts of best practices, more justification for the need for increased quantitative literacy, and discussions on the factors that contribute to the current replication crisis. However, some additional points of clarification and expansion on key points are needed.

Reviewers' comments:

Reviewer #1:

The authors strenghtened the paper by addressing in detail the issues raised during the first review.

Reviewer #2:

This commentary addresses an important topic for science- education on quantitative literacy and the implications for reproducibility and transparency across laboratories. The authors have done an excellent job of conveying the importance of training in quantitative literacy, and the T32 initiative in hosting a retreat to address this component of training is commendable.

The commentary, however, could benefit from concrete examples of activities (coupled with the pros/cons, opportunities for improvement). Clear and transparent descriptions of successful versus less ideal activities would help others develop successful retreat agendas. Discrete recommendations would also add to the paper- for example what discussion group size was considered optimal, at what group size did active participation noticeably decrease? The text eludes to an effect of group size on learning, but does not provide any insight into what an ideal number of participants could be. With respect to the post retreat survey, and the idea that "organization of the breakout rooms could be improved by providing the leaders with a list of discussion points related to the topic," examples of activities, and suggestions for improvement of structure, would also be informative for others in future retreat planning. Clear examples should be provided throughout the paper, with respect to improving the efficacy of training retreats. Finally, it is not clear how inclusion of a person with lived experience advances understanding of quantitative literacy. Educating scientists on the real-world implications of injury is an equally, if not more, important retreat goal, but how participants felt that this would benefit the objectives of this program isn't obvious. This needs to be clarified.

## Author Response

Rebuttal Letter:

Responses to Reviewers' Comments Manuscript NO. eN-COM-0027-25 We are grateful for the opportunity to resubmit for publication the revised version of eN-COM-0027-25 "Incorporating Quantitative Literacy into a T32 Retreat: Lessons and Considerations from Experience" for consideration in eNeuro. We thank the reviewers for their time and detailed feedback. The reviewers agreed that the manuscript has the potential to impact future training activities by emphasizing active learning. We value the constructive criticisms of the reviewers which will make this paper more robust and relevant. We have addressed each of their concerns as outlined below. Changes are identified in bold throughout the manuscript.

Reviewer #2:

1) The commentary could benefit from concrete examples of activities with respect to improving the efficacy of training retreats.

We have added multiple examples throughout the commentary of activities that could improve the efficacy of training retreats. We have added examples of topic-centered handouts to help facilitate breakout rooms and describe splitting breakout rooms into subgroups to foster discussion. We also provide suggestions for how to incorporate student submitted questions to improve the efficacy of a workshop session.

2) Discrete recommendations would add to the paper, such as the optimal discussion group size, and at what group size active participation noticeable decreases.

In the Best Practices and Guidance for Retreat Planning section, we have added discussion about the ideal group size for discussion. We provide recommendations for optimal size based off our experiences, and have added at what size we noticed a decrease in group participation.

3) It is not clear how the inclusion of a person with lived experience advances understanding of quantitative literacy.

We have added clarification to the Best Practices and Guidance for Retreat Planning section on how including people with lived experience advances quantitative literacy. We discuss how the importance of hearing from individuals with lived experience helps to reify rigorous science that is critical for treatment development.
